# Supplementary material for: Catalytic Conversion of Glucose into Levulinic Acid Using 2-Phenyl-2-Imidazoline Based Ionic Liquid Catalyst
Source: Molecules. 2021 Jan 12;26(2):348. doi: 10.3390/molecules26020348 (PMC7827230; doi:10.3390/molecules26020348)
Supplement: Supplementary file 1 [file molecules-26-00348-s001.pdf]

Catalytic Conversion of Glucose into Levulinic Acid Using 2-Phenyl-2-Imidazoline Based Ionic Liquid Catalyst

Komal Kumar, Mukesh Kumar and Sreedevi Upadhyayula *

Department of Chemical Engineering Indian Institute of Technology Delhi, New Delhi, Hauz Khas, New Delhi 110016, India. komalkumar924@gmail.com (K.K.); mukeshbahl924@gmail.com (M.K.)

*Corresponding author: sreedevi@chemical.iitd.ac.in; Tel.: +91 11 26591083, Fax: +91 11 26591120

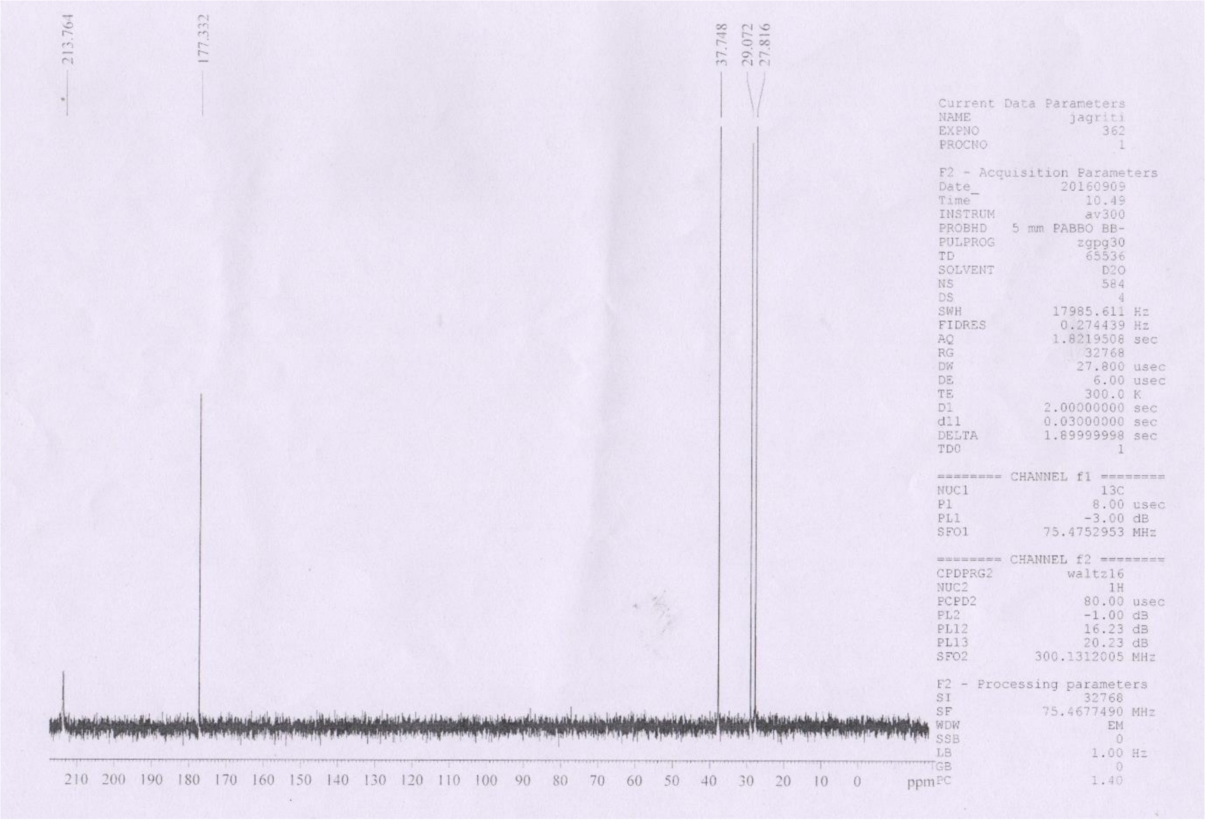

Figure S1. ^{13}C NMR Spectrum of the purified LA.

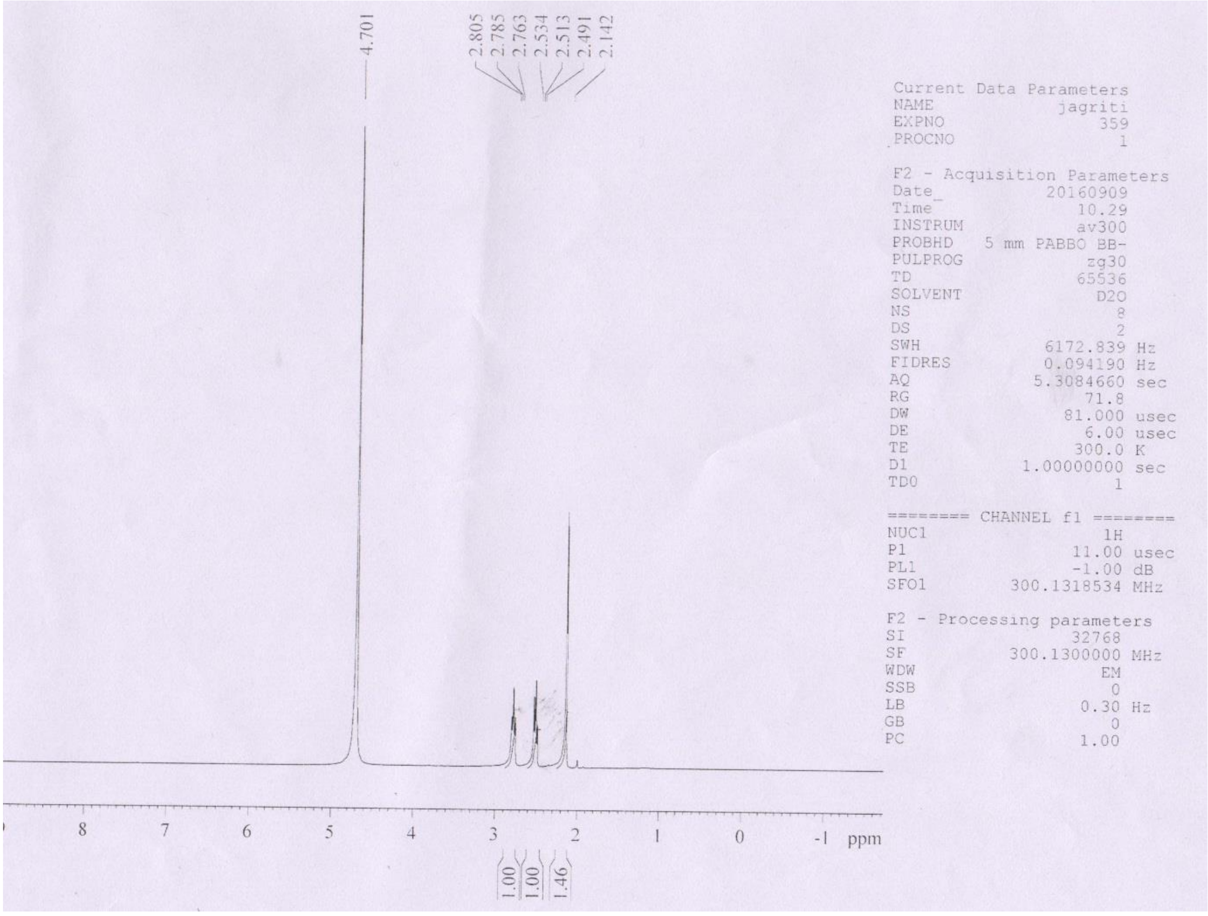

Figure S2. ^{1}H NMR spectrum of purified LA.
